# The Comparison of Semen Collection in Electroejaculation, Rectal Massage and Combination of Both Methods in the Critically Endangered Malayan Pangolin, *Manis javanica*

**DOI:** 10.3390/ani10111948

**Published:** 2020-10-23

**Authors:** Reza Tarmizi, Yap Keng Chee, Symphorosa Sipangkui, Zainal Zahari Zainuddin, Wan-Nor Fitri

**Affiliations:** 1 Borneo Rhino Alliance, Sabah, Malaysia, c/o Faculty of Sciences and Natural Resources, Universiti Malaysia Sabah, Kota Kinabalu 88400, Sabah, Malaysia; reza2727@gmail.com (R.T.); kcyap.vet.upm@gmail.com (Y.K.C.); zainalz.bora@gmail.com (Z.Z.Z.); 2 Sabah Wildlife Department, Tingkat 4, Blok B, Wisma MUIS, Kota Kinabalu 88100, Sabah, Malaysia; symphorosa.sipangkui@sabah.gov.my; 3 Theriogenology and Cytogenetics Laboratory, Department of Veterinary Clinical Studies, Faculty of Veterinary Medicine, Universiti Putra Malaysia (UPM), Seri Kembangan 43400, Selangor, Malaysia

**Keywords:** biobank, wildlife, illegal wildlife trade, assisted reproductive technology

## Abstract

**Simple Summary:**

Semen collection is one of the first few foundational steps towards Assisted Reproductive Technology (ART), however it is rarely conducted in the Malayan pangolin, *Manis javanica.* Semen was successfully retrieved in pangolins mainly via electroejaculation with the animal under general anesthesia, though facing a minor risk of urine contamination. The objective of this study is to report the success of semen collection with three different collection methods: electroejaculation, rectal massage and a combination of both methods in pangolins. The semen characteristics of the species are described in this study and the good semen qualities suggest future potential uses for ART. This study has found no difference between methods of semen collection. Captive and confiscated animals only differed in the percentages of live spermatozoa with overall good semen characteristics. The results from this study suggest that gamete recovery and biobank are feasible in the Malayan pangolin, which is essential to the management and conservation of this endangered species.

**Abstract:**

This article describes the semen characteristics from different collection methods between captive and confiscated Malayan pangolins, *Manis javanica*. Semen was collected from 15 pangolins; two captive and 13 confiscated individuals at the mean weight of 9.36 ± 1.94 kg. The three semen collection methods employed were electroejaculation, rectal massage and a combination of both techniques. The semen characteristics (mean ± standard deviation) of the Malayan pangolin are volume (73.75 ± 144.57 µL), pH (7.63 ± 0.53), spermatozoa concentration (997.19 ± 728.98 × 10^6^ /mL), total motility (59.60% ± 30.00%), progressive motility (48.95% ± 30.93%), mass motility (3.50 ± 1.50) and live spermatozoa (80.25% ± 13.45%). There was no significant difference in semen characteristics between the three collection methods. The percentages of live spermatozoa were significantly different, suggesting better samples from captive compared to confiscated animals. However, there was no significant difference in spermatozoa kinetics between the captive and confiscated samples, suggesting the potential of utilizing confiscated individuals for gamete recovery to conserve the genetic pool of pangolins. All three methods of semen collection were successfully performed in pangolins and should be considered; however, electroejaculation remains the most consistent method of obtaining semen from the species.

## 1. Introduction

The Malayan pangolin (*Manis javanica*) is a critically endangered scaly anteater, with a home range in South East Asia. This nocturnal mammal, belonging to the order Pholidota, is one of the eight extant species of pangolins, and is closely related to the order Carnivora [1,2]. The population of Malayan pangolins in Peninsular Malaysia and throughout the islands of Borneo is severely under the threat of local extinction due to the illegal wildlife trade [3]. The trend of poaching activities is also concerning, with an estimated 192,567 pangolins seized between 1999 and 2017 [4]. Despite making the headlines during the recent coronavirus outbreak COVID-19 [5], pangolins are still being trafficked and the demand is still on the rise, as evidenced by the recent pangolins raid in Malaysia [6]. The critically endangered status of the Malayan pangolin and the increasing number of confiscated individuals suggest the need for a review of the ex-situ management program. The sudden increase in the availability of genetic reserve makes it necessary to consider the development of a gamete storage technique, and to increase the capacity of biobanking.

The effort of establishing assisted reproductive technology (ART) in Malayan pangolins is still at its infancy, despite being listed as critically endangered, at par with the Malayan tiger and Sumatran rhinoceros [7], the latter facing extinction in Malaysia recently [8]. The breeding and conservation of the species are of the utmost priority, since the three Malayan pangolin lineages are different and should be managed as distinct conservation units [9]. One of the best available tools to maximize the breeding of wildlife in ex situ conservation is ART [10]. However, it is integral to collate fundamental reproductive data to develop the right set of tools for the application of ART [11]. Semen collection and evaluation is one of the earlier required techniques to be established in pangolins, both for breeding soundness evaluation and ART development. Therefore, it is important to evaluate the reproductive health and establish good semen collection techniques for the future captive breeding program of the species.

The reproductive behavior [12] and the success of captive breeding in the Malayan pangolin have been reported [13]. The reproductive biology in male Malayan pangolins has also been explored recently [14,15]. The success of breeding the species in captivity suggests that the standard of husbandry and fertility of pangolins in captivity are satisfactory. However, the effect of acute and prolonged stress from poaching on the reproduction and fertility of pangolins is still being questioned [16]. Captive pangolins are not meant for domestication [17], however confiscated individuals were kept in captivity because of the challenges of reintroducing the pangolins back into the wild [18]. Several thousand pangolins are affected by the illegal wildlife trade [19], and semen collection will improve utilization and gamete banking through ART. Reproduction has been outlined, together with artificial habitat, nutritional [20] and disease control, as the main challenges for the captive breeding program in pangolins [21]. Yet studies on reproductive biology and ART in pangolins are still scarce. The technique of ART in wildlife tends to be species-specific due to each species’ unique reproductive anatomy and physiology [22], making it urgent to take advantage of the confiscated individuals in a specific reproductive study of the Malayan pangolin.

Optimum semen collection methods are important to ensure success in ART. Various semen collection methods were described in wildlife, and innovation in semen collection is still being discovered [23], although many techniques have been adapted from domestic animals [10]. Yet the application, knowledge and success in different species varies, which suggests the need for a species-specific investigation. Semen collection by electroejaculation used to be the only means of achieving semen collection in wildlife and untrained animals. This highlights the importance of adding variation to the technique of semen collection by reducing the electrical stimulant given to the animal [24]. Reproductive health may be an important secondary welfare indicator [25] that perhaps is more sensitive than initially thought in pangolins. This article reports successful semen collection using electroejaculation, rectal massage and a combination of both in captive and confiscated Malayan pangolins on the island of Borneo.

## 2. Materials and Methods

### 2.1. Animals

There were 15 male pangolins involved in this study—2 captive and 13 confiscated pangolins. All 15 pangolins were originally confiscated from the illegal wildlife trade. The pangolins which were considered as the captive group were the animals under the care of the Lok Kawi Wildlife Park. The two captive pangolins were acclimatized in a habitat made for pangolins within the wildlife park for 3 months before the semen collection procedure took place between August 2018 and January 2019. The remaining 13 confiscated pangolins were from two batches of raided specimens from two areas of Sabah, which were Tamparuli and Keningau, in February of 2019. Semen collections were performed on the confiscated group once approved by the Sabah Wildlife Department (SWD), within a week of confiscation. The minimum body weight criteria to be considered as sexually mature and clinically fit were being above 7kg with a good body condition score. The mean body weight of all the pangolins was 9.36 ± 1.94 kg. The semen collection procedure was always overseen by veterinarians from SWD and Borneo Rhino Alliance (BORA), and this research was approved by the Wildlife Department under the approval code JHL(HQ)400-9/82 JLD 9.

### 2.2. Semen Collection

Semen was collected from all 15 individual pangolins, constituting 20 collection attempts using the three methods of semen collection: electroejaculation, rectal massage and a combination of electroejaculation and rectal massage. The semen collection was attempted in confiscated pangolins (*N* = 13) and in captive pangolins (*N* = 2); twice and five attempts in each animal respectively (Table 1). The pangolins were all generally anaesthetized with a combination of (MKB) Medetomidine (Dorbene vet 1mg/mL, Zoetis), Ketamine (Ketamil 100mg/mL, Ilium) and Butorphanol (S6 50mg/mL, Kyron), all given intramuscularly at the dosage of 0.1mg/kg, 5mg/kg and 0.1mg/kg, respectively. At the end of the procedure, all the animals were given an antidote of Atipamezole (Atipamezole, 5mg/mL, Ilium), intramuscularly five times the dose of Medetomidine, and Naltrexone (S4 40mg/mL, Kyron), intramuscularly two times the dose of Butorphanol. 

**Figure 1:**
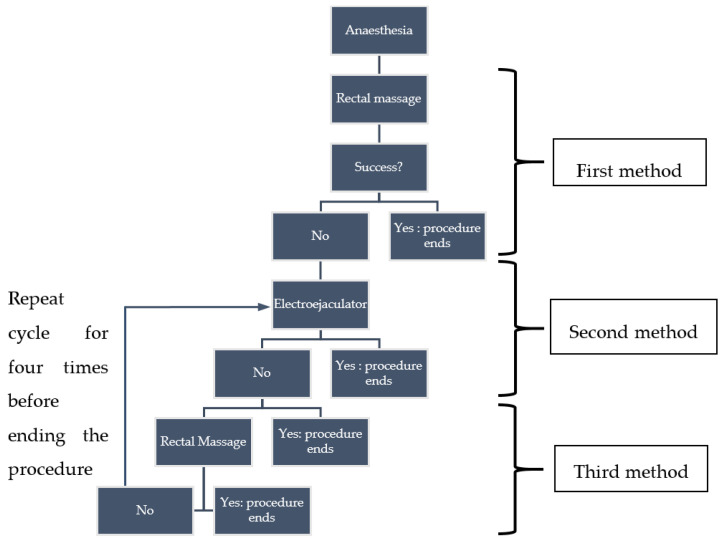
Pangolin semen collection flow and methods. Success in each initial method prevents the continuation of further semen collection methods until the procedure ends at the fourth cycle of electrical stimulation.

The semen collection methods were performed according to this order: (1) rectal massage, (2) electroejaculator and (3) combination of both methods. The success of semen collection during the rectal massage, manual feces evacuation and probe placement prevent the continual attempt of the following second and third methods. In an event whereby the semen was not obtained by rectal massage, the electroejaculator procedure followed. Semen was considered as obtained by electroejaculator when ejaculates were acquired during electrical stimulation. Finally, semen was considered as obtained by a combination of both methods when the ejaculates were acquired between the electroejaculator cycle, when the rectal massage was performed with no electrical stimulation applied. The flow of the semen collection procedure is simplified in Figure 1. Following the three-step semen collection protocol, the attempts were made 17 times with the electroejaculator, twice with the rectal massage and once with the combination of both methods. 

The semen collection procedure was preceded by the evacuation of feces and the placement of the rectal probe using a Seager Model 14 electro-ejaculator (Dalzell USA Medical Systems, The Plains, VA, USA), equipped with 1.0 cm diameter rectal probe. During the rectal manipulation, the flow of semen can be observed occasionally from the penile opening, thus the procedure was followed by the massaging of the ventral rectal wall. The digital massage of the rectal wall (rectal massage) was accompanied by a gentle massage of the ventral abdomen in a craniocaudal motion towards the accessory glands and vas deferens to facilitate the flow of semen. Following the rectal and abdominal massages, the electroejaculation method was followed up in four cycles until the collection ended, starting from 3 volts up to a maximum of 7 volts in a total of 4 stimulations, with 10 cycles for each stimulation. The rectal and abdominal massage was performed between the electrical stimulation.

### 2.3. Semen Evaluation

The semen characteristics evaluation was mainly based on a standard in pangolins [26]. However, the standard parameters for semen description were slightly modified based on standards established in domestic animals and wildlife [27,28,29]. Semen volume was estimated based on the droplets of semen, each droplet with the size of 0.5 mm in diameter to be averaged at 15 µL/drop. Semen concentration was determined using a Neubaeur haemocytometer counting chamber (VWR International, Lutterworth, Leicestershire, UK) [30]. Semen was maintained in a water bath at 37.5 °C before semen motility assessment. Total motility and progressive motility were estimated under a phase-contrast microscope at 400x magnification (Olympus Corporation, Tokyo, Japan) under x40 objective, by two experienced veterinary laboratory technicians, blinded on the condition of the animals and independent of each other in order to obtain an unbiased evaluation and an average value of the sperm motility. The mass motility in pangolins were established based on standards in domestic animals [31,32]. Semen was also subjected to live–dead analysis, diluted with one-part semen and three-part Eosin Nigrosin stain and thin-smeared on a glass slide. The pH of semen was determined by using pH strips as an indication for urine contamination (Whatman^®^ pH indicator paper strip, Marlborough, MA, USA). 

### 2.4. Statistical Analysis

The ejaculates collected were subjected to analysis using the statistical package IBM SPSS Statistics 20, and values were reported as mean ± standard deviation. Analysis of variance was used to identify a significant difference between ejaculates from different individuals, rejected and accepted samples, collection methods, and between captive and confiscated pangolins at *p* < 0.05. Frequency distribution tables were also used to obtain and report on the efficiency of the methods used.

## 3. Results

The consistency of semen in pangolins is sticky, slimy, thick and creamy, and appears white to a slight hue of a yellowish tinge. Semen was successfully collected in all three methods 17/20 times (85%), by electroejaculator 2/20 times (10%), and by rectal massage 1/20 times (5%) in the combination methods. A minute semen volume (73.75 ± 144.57µL), which was highly concentrated (997.19 ± 728.98 × 10^6^ /mL) and had moderate total motility (59.60 ± 30.00), progressive motility (48.95 ± 30.93) and mass motility (3.50 ± 1.50), as well as a high live spermatozoa count (80.25 ± 13.45), was observed in pangolins.

There was no significant difference between individual animals, thus semen characteristics were pooled. Mean semen characteristics between the urine-contaminated and urine-free samples were reported in Table 2. The high volume of ejaculates showed results of a mixture with urine; 660 µL and 200µL, low spermatozoa concentrations of 381.25 × 10^6^ /mL and 100 × 10^6^ /mL, a lower pH of 6.25 and zero spermatozoa motility in this study was confirmatory of urine contamination in the two collection attempts using an electroejaculator. The semen characteristics between different collections are reported in Table 3. However, there was no significant difference between semen collection methods. Semen collection by rectal massage and a combination of methods was successfully attempted in captive populations only, meanwhile the use of an electroejaculator was necessary for all the confiscated population. The semen characteristics of captive and confiscated pangolins are reported in Table 4, with a significant difference between the live percentages of spermatozoa.

## 4. Discussion

The first known attempts at semen collection, describing the different methods of collection and semen characteristics in pangolins, are reported in this study. There were reports of spermatozoa morphology in pangolins [15,26,33,34], yet semen collection and the characteristics within the genus, species and order remain to be elucidated. The pangolins are closely related phylogenetically with the order of Carnivora [35], prompting a potential comparison across the order due to the lack of available data. Although some semen characteristics in pangolins were similar when compared to the wild felid [36], the semen volume, concentration and viability appeared to be different. In contrast, the semen characteristics in brown bears seemed to be superior in terms of total motility [37]. The comparison of semen characteristics across distinct species, due to physically different anatomies, physiologies and the sheer size alone, could explain the variation, especially in the semen volume. Thus, it was suggested to conduct the study of semen characteristics in a species-specific manner in order to overcome the differences and specific challenges to ART application [38]. It is also understood that a comparison of semen characteristics even between the most closely related orders, Pholidota and Carnivora, fell short in many ways, highlighting the importance of this study to the reproductive knowledge of pangolins. 

Pangolin semen characteristics are suitable for future work in ART. The semen has a thick and slimy consistency, which does not take well to being aspirated with single-channel micropipette fitted with micropipette tips (0.5–10 µL, Eppendorf Research^®^ plus G, single-channel, medium grey epT.I.P.S^®^). Thus, the method of estimating semen droplets with highly viscous ejaculates using droplets size was the best option in the field to estimate the semen volume after using a mechanical micropipette in wildlife [39]. There are various factors related to the success of artificial insemination in wildlife. Spermatozoa kinetics is deemed one of the factors which is important for the outcome of fertility [40]. The semen kinetics (total motility, progressive motility and mass motility) in pangolins are good, though faced with the challenge of the minute volume of semen yield, the ejaculates can further be extended using a semen extender. There is high potential in using ART in pangolins with the current semen quality, as attempts in ART with even lower semen parameters were found to be successful for in wildlife [41]. Artificial insemination is yet to be performed in pangolins; however, successful captive breeding in pangolins suggests that the animals fare well in captivity, and captive breeding is possible [21]. 

The overall information on semen collection and reproductive biology in pangolins is limited. The efficiency between the three collection methods still proves that the electroejaculator remains the most consistent method to obtain semen from the species. The small sample size in this study limits the explanation of the observed three methods of semen collection in the captive populations. However, the experience of semen collection via rectal massage and the combination of rectal massage and electroejaculator usage suggests that these two methods are also possible in pangolins. These findings offer further physiological and welfare understanding of the effects of the illegal wildlife trade on reproduction, although stressors are known to affect reproduction in wildlife [42]. Digital massage alone has been a successful tool in collecting semen from various species of wildlife, such as crocodiles, Bali cattle and endangered nonhuman primates [43,44,45]. In this study, general anesthesia provides better welfare to the animal as it is painless and less stressful [46]. Apart from that, the drug also has an alpha-adrenergic effect on the smooth muscle involved with semen release, allowing the flow of semen in the reproductive tract to the penile tip [47]. The findings were similar in the use of anesthesia in other wild mammals, using the xylazine combination under general anesthesia, in which the semen can sometimes be collected readily on the penile tip with the manipulation and positioning of the electroejaculator probe as an effect of immobilization [48]. However, it was found that medetomidine has 10 times more specificity towards the alpha-2-receptors than xylazine, indicating medetomidine as the better drug for spermatozoa recovery [49]. Digital massage of the rectal for semen collection in the sedated animals was also observed in other wildlife, together with the aid of gravitational pull through the positioning of the animal on a raised platform [50]. Semen collection by electroejaculator usage poses the risk of urine contamination, as observed in this study. Although electorejaculator usage was consistent in yielding semen in pangolins, the methods are known to carry the risk of urine contamination in other mammals [51,52]. Urine contamination can also be reflective in the increased volume of ejaculates due to urine contamination, the decreased pH and motility of the spermatozoa [53], the occurrence of zero spermatozoa motility as well as the pH reduction in the two samples derived in our case. The higher live spermatozoa count in urine-contaminated samples compared with urine-free samples suggests that the initial quality of semen collected from urine-contaminated samples could potentially be better. However, this potential was greatly reduced due to urine contamination. After the urine-contaminated samples were removed, the semen characteristics of pangolins showed improved kinetic motilities and better live spermatozoa counts. The results from this study propose that it would be possible and promising to cryopreserved pangolin semen for future use in ART.

The important factors of reproduction between captive and confiscated animals are health and stress. Confiscated pangolins are known to be heavily infested with parasites, inflicted with multiple traumatic wounds related to transport and handling, as well as hunger-associated complications [54]. These lesions are associated with survival more than reproductive potential, especially when considering a release program. However, the long-term impacts of poaching were observed to pose an impact on the stress physiology and reproductive output in female African elephants [55]. These observations could likely be reproducible in pangolins, and the effect would be more immediate given the shorter lifespan. This study suggests that the difference in semen characteristics is in the live spermatozoa percentage between the captive and confiscated animals. Health evaluation of the confiscated animal is of the utmost importance to the welfare of the animal, and has been described in detail in pangolins [56]. The reproductive status may be an indirect indicator of the wellbeing of pangolins in captivity, similar to other captive mammals [56]. The minor difference in semen quality in captive and confiscated pangolins suggests that it can adapt well in captivity, given the right husbandry. Semen collection for the potential use of gamete storage and cryopreservation in the confiscated individuals was also shown to be possible. Therefore, semen collection should be considered as part of a routine procedure to improve conservation efforts in the Malayan pangolin. 

## 5. Conclusions

Semen collection attempts were successful using electroejaculation, rectal massage and a combination of rectal massage and electroejaculation in pangolins. Electroejaculator usage remains the most consistent method of semen collection, however success in rectal massaging and the combination of both methods is proposed to be possible and advantageous in the species. The semen quality is better in the captive compared to the confiscated animals, however without any difference in the spermatozoa’s kinetics. The success of semen collection for gamete storage and cryopreservation in confiscated individuals in this study suggests that the procedure should be considered as part of routine practice to improve the conservation effort in this species.

## Figures and Tables

**Table 1:** Semen collection details of individual pangolins.

No.	Pangolin ID	Location	Captive	Confiscated	Collection Attempts		
					RM	EE	EE + RM
1.	000728DD97	Lok Kawi Wildlife Park	•			2	
2.	00074FD17F	Lok Kawi Wildlife Park	•		2	2	1
3.	EX1	Tamparuli		o		1	
4.	EX2	Tamparuli		o		1	
5.	EX3	Tamparuli		o		1	
6.	EX4	Tamparuli		o		1	
7.	EX5	Tamparuli		o		1	
8.	EX6	Tamparuli		o		1	
9.	EX7	Tamparuli		o		1	
10.	JG1	Keningau		o		1	
11.	JG2	Keningau		o		1	
12.	JG3	Keningau		o		1	
13.	JG4	Keningau		o		1	
14.	JG5	Keningau		o		1	
15.	JG6	Keningau		o		1	

RM: rectal massage, EE: electroejaculator.

**Table 2:** Mean semen characteristics in urine-contaminated and urine-free samples in pangolins.

Semen Characteristics	Mean ± SD (*n* = 20)	Mean Semen Characteristics	
		Urine Contaminated (*n* = 2)	Urine Free (*n* = 18)
Volume (µL)	73.75 ± 144.57 (5.00–660.00)	430.00 ± 325.27 ^a^ (200.00–660.00)	34.17 ± 23.34 ^b^ (5.00–90.00)
pH	7.63 ± 0.53 (6.25–8.00)	6.25 ± 0.00 ^a^ (6.25)	7.78 ± 0.26 ^b^ (7.50–8.00)
Spermatozoa concentration (×10^6^ /mL)	997.19 ± 728.98 (100.00–2541.00)	240.63 ± 198.87 (100.00–381.25)	1081.25 ± 718.87 (107.50–2541.00)
Total motility (%)	59.60 ± 30.00 (0.00–90.00)	0.00 ± 0.00 ^a^ (0.00)	66.22 ± 23.27 ^b^ (15.00–90.00)
Progressive motility (%)	48.95 ± 30.93 (0.00–90.00)	0.00 ± 0.00 ^a^ (0.00)	54.39 ± 27.50 ^b^ (7.00–90.00)
Mass motility (0–5)	3.50 ± 1.50 (0.00–5.00)	0.00 ± 0.00 ^a^ (0.00)	3.89 ± 0.96 ^b^ (2.00–5.00)
Live spermatozoa (%)	80.25 ± 13.45 (36.10–97.50)	92.50 ± 0.71 (92.00–93.00)	78.89 ± 13.51 (36.10–97.50)

Values are mean ± SD (ranges). Different superscripts between column differ significantly, *p* < 0.05.

**Table 3:** Mean semen characteristics between different collection methods in pangolins.

Semen Characteristics	Mean Semen Characteristics		
	Rectal Massage (*n* = 2)	Electroejaculator (*n* = 17)	Combination (*n* = 1)
Volume (µL)	42.50 ± 3.54 (40.00–45.00)	80.00 ± 156.63 (5.00–660.00)	30.00
pH	7.50 ± 0.00 (7.50)	7.65 ± 0.57 (6.25–8.00)	7.50
Spermatozoa concentration (×10^6^ /mL)	1247.95 ± 38.25 (1220.90–1275.00)	944.73 ± 781.44 (100.00–2541.00)	1387.50
Total motility (%)	90.00 ± 0.00 (90.00)	54.82 ± 30.05 (0.00–90.00)	80.00
Progressive motility (%)	85.00 ± 7.07 (80.00–90.00)	43.47 ± 30.19 (0.00–90.00)	70.00
Mass motility (0–5)	5.00 ± 0.00 (5.00)	3.24 ± 1.48 (0.00–5.00)	5.00
Live spermatozoa (%)	93.20 ± 6.08	78.27 ± 13.55 (36.10–93.00)	88.00
Collection success (%)	10	85	5

**Table 4:** Mean semen characteristics in captive and confiscated pangolins.

Semen Characteristics	Mean Semen Characteristics in Captive or Confiscated Animal	
	Captive (*n* = 7)	Confiscated (*n* = 13)
Volume (µL)	134.29 ± 232.06 (30.00–660.00)	41.15 ± 53.63 (5.00–200.00)
pH	7.32 ± 0.47 (6.25–7.50)	7.79 ± 0.50 (6.25–8.00)
Spermatozoa concentration (×10^6^ /mL)	995.38 ± 426.39 (381.25–1387.50)	998.16 ± 866.30 (100.00–2541.00)
Total motility (%)	65.00 ± 33.54 (0.00–90.00)	56.69 ±28.92 (0.00–90.00)
Progressive motility (%)	59.29 ± 34.45 (0.00–90.00)	43.38 ± 28.74 (0.00–80.00)
Mass motility (0-5)	3.86 ± 1.86 (0.00–5.00)	3.31 ± 1.32 (0.00–5.00)
Live spermatozoa (%)	89.94 ± 4.52 ^a^ (82.70–97.50)	75.03 ± 13.85 ^b^ (36.10–93.00)

Values are mean ± SD (ranges). Different superscripts between column differ significantly, *p* < 0.05.
